# Longitudinal Associations between 24-h Movement Behaviors and Cardiometabolic Biomarkers: A Natural Experiment over Retirement

**DOI:** 10.1249/MSS.0000000000003415

**Published:** 2024-02-26

**Authors:** KRISTIN SUORSA, TUIJA LESKINEN, NIDHI GUPTA, LARS L. ANDERSEN, JESSE PASANEN, PASAN HETTIARACHCHI, PETER J. JOHANSSON, JAANA PENTTI, JUSSI VAHTERA, SARI STENHOLM

**Affiliations:** 1 Department of Public Health, University of Turku and Turku University Hospital, Turku, FINLAND; 2 Centre for Population Health Research, University of Turku and Turku University Hospital Turku, Turku, FINLAND; 3 Department of Musculoskeletal Disorders and Physical Work Load, National Research Centre for the Working Environment, Copenhagen, DENMARK; 4 Department of Medical Sciences, Occupational and Environmental Medicine, Uppsala University, Uppsala, SWEDEN; 5 Occupational and Environmental Medicine, Uppsala University Hospital, Uppsala, SWEDEN; 6 Clinicum, Faculty of Medicine, University of Helsinki, Helsinki, FINLAND; 7 Research Services, Turku University Hospital and University of Turku, Turku, FINLAND

**Keywords:** ACCELEROMETER, SLEEP, SEDENTARY TIME, PHYSICAL ACTIVITY, COMPOSITIONAL DATA ANALYSIS, RETIREMENT, AGING, CARDIOMETABOLIC HEALTH

## Abstract

**Introduction:**

Physical activity, sedentary behavior, and sleep, that is, 24-h movement behaviors, often change in the transition from work to retirement, which may affect cardiometabolic health. This study investigates the longitudinal associations between changes in 24-h movement behaviors and cardiometabolic biomarkers during the retirement transition.

**Methods:**

Retiring public sector workers (*n* = 212; mean (SD) age, 63.5 (1.1) yr) from the Finnish Retirement and Aging study used a thigh-worn Axivity accelerometer and filled out a diary to obtain data on daily time spent in sedentary behavior (SED), light physical activity (LPA), and moderate-to-vigorous physical activity (MVPA), and sleep before and after retirement (1 yr in-between). Cardiometabolic biomarkers, including LDL-cholesterol, HDL-cholesterol, total/HDL-cholesterol ratio, triglycerides, C-reactive protein, fasting glucose, and insulin, were measured. Associations between changes in 24-h movement behaviors and cardiometabolic biomarkers were analyzed using compositional robust regression and isotemporal substitution analysis.

**Results:**

Increasing LPA in relation to remaining behaviors was associated with an increase in HDL-cholesterol and decrease in total/HDL-cholesterol ratio (*P* < 0.05 for both). For instance, reallocation of 30 min from sleep/SED to LPA was associated with an increase in HDL-cholesterol by 0.02 mmol·L^−1^. Moreover, increasing MVPA in relation to remaining behaviors was associated with a decrease in triglycerides (*P* = 0.02). Reallocation of 30 min from SED/sleep to MVPA was associated with 0.07–0.08 mmol·L^−1^ decrease in triglycerides. Findings related to LDL-cholesterol, C-reactive protein, fasting glucose, and insulin were less conclusive.

**Conclusions:**

During the transition from work to retirement, increasing physical activity at the expense of passive behaviors was associated with a better lipid profile. Our findings suggest that life transitions like retirement could be utilized more as an optimal time window for promoting physical activity and health.

Regular physical activity reduces the risk of type 2 diabetes and cardiovascular diseases (1,2), which are leading causes of death worldwide (3). However, time spent on physical activity constitutes only a small proportion of a 24-h day, with the remaining time spent in sleep and sedentary behavior (SED). Studies have usually treated these as independent behaviors and concluded that not only increasing moderate-to-vigorous intensity leisure-time physical activity but also increasing light-intensity physical activity (LPA) and reducing SED improve cardiometabolic health (4,5). Regarding sleep, both short (<7 h) and long durations (>9 h) have been associated with a higher risk of cardiovascular disease and type 2 diabetes (6–8).

In contrast to common analytical approaches, research performed in recent years recognizes that sleep, SED, LPA, and moderate-to-vigorous physical activity (MVPA), often referred together as 24-h movement behaviors, are not independent from each other, because it is impossible to increase one behavior, for instance, MVPA, without reducing at least one other behavior (9,10). Health effects related to MVPA may also differ depending on the compensatory effect of increasing MVPA, that is, which behavior or behaviors is/are being replaced (11,12). Ignoring the codependency between the 24-h movement behaviors may result in misleading findings (9,13). Thus, this requires adopting a comprehensive approach that addresses such codependency between movement behaviors.

One such approach is compositional data analysis (CoDA) (9,10,14). The CoDA addresses codependency between components, in this case 24-h movement behaviors, by treating components relative to each other as logratios (14). Recent cross-sectional studies applying CoDA to 24-h time spent on movement behaviors in adults have mainly indicated that more physical activity, especially MVPA in relation to sleep and SED, is associated with lower triglycerides (9,15–17), higher HDL-cholesterol (9,16–18), and lower blood glucose and insulin (9,16,17,19). The main limitation in these previous studies applying CoDA is the reliance on cross-sectional data; thus, it remains unknown how actual within-individual changes in 24-h movement behaviors are associated with changes in cardiometabolic biomarkers.

Life transitions such as retirement can be utilized as natural experiment settings to examine how within-individual changes in 24-h movement behaviors are associated with changes in health outcomes (20). We have previously reported that retirement changes the composition of a 24-h day toward more sleep at the expense of physical activity (21). Also SED changes during the retirement transition, so that those retiring from manual occupations increase their SED, leading to substantial decreases in physical activity, whereas nonmanual workers sleep more at the expense of less SED (21). These changes in 24-h movement behaviors associate with changes in obesity indicators (22). We observed that increasing the proportion of sleep at the expense of less physical activity, especially less MVPA, was associated with an increase in body mass index and waist circumference (22). Moreover, previous studies have suggested that the transition to retirement is also associated with negative changes in cardiometabolic health (23,24), but it is unknown whether retirement-induced changes in 24-h movement behaviors are associated with changes in cardiometabolic biomarkers.

The aim of this study was to examine how changes in 24-h movement behaviors are associated with changes in cardiometabolic biomarkers over 1 yr during the transition from work to retirement.

## METHODS

### Study design and participants

The study population consisted of participants from the Finnish Retirement and Aging Study (FIREA), an ongoing longitudinal cohort study of older adults in Finland established in 2013. Details of the design and implementation of the FIREA study have been reported elsewhere (25). Shortly, participants were first contacted 18 months before their estimated retirement date by sending them a questionnaire. After responding to the questionnaire, Finnish-speaking participants with estimated retirement date between 2017 and 2019, who lived in Southwest Finland and were still working, were invited to participate in the clinical substudy (*n* = 773). Of them, 290 agreed to participate. Thereafter, study participants were followed up with annual measurements including questionnaires, and clinical and accelerometer measurements. To determine the timing of retirement, the actual retirement day was inquired during each phase of the data collection, and this information was used to determine preretirement and postretirement measurements.

Of the clinical substudy participants, 241 took part in accelerometer and laboratory measurements before and after the transition to full-time statutory retirement, with on average 1 yr in between the measurements. We excluded participants who had less than 3 valid accelerometer measurement days before and/or after retirement (*n* = 29), leaving 212 participants to the analytical sample (Supplemental Fig. 1, Supplemental Digital Content 1, Flow chart for the selection of the study population, http://links.lww.com/MSS/C986).

### Assessment of 24-h movement behaviors before and after retirement

Participants wore a triaxial accelerometer Axivity AX3 (Axivity Ltd, Newcastle, UK) on the thigh and filled out a daily diary to estimate 24-h movement behaviors: sleep, SED, LPA, and MVPA. Detailed description of the measurement protocol is reported elsewhere (22,26). The accelerometer was fastened to the skin of the front of the right thigh, midway between the iliac crest and the upper line of patella with adhesive waterproof film dressing during the clinical visit. Participants were instructed to wear the accelerometer at all times, 24 h·d^−1^. Before retirement, participants were asked to wear the accelerometer at least 4 d and nights, including at least 2 workdays and 2 days off, and after retirement at least 4 d and nights. During the measurement time, participants were instructed to perform a reference measurement in a standing upright position for 15 s each day and record the time of reference measurement, and also the waking time, time of going to bed, and start and end of the work interval on workday.

Data from the accelerometers were downloaded through Open Movement software (version 1.0.0.37; Open Movement, Newcastle University, Newcastle upon Tyne, UK). The raw data were processed to determine 24-h movement behaviors using a customized MATLAB program, ActiPASS (version 0.80) (27), an automatized version of Acti4 (28,29), which determines the type and duration of different activities and body postures with a high sensitivity and specificity (28,29). Measurement period was restricted to days between the first and last dates and time recorded in the daily log. Nonwear time was detected using algorithm in the ActiPASS (≥60 min periods without movement) (28). The measurement day was determined from midnight to midnight, and a valid measurement day was defined as a day with at least 10 h of wear time during waking hours and daily log-determined waking and bedtimes.

ActiPASS was used to determine time spent sitting, lying (30), standing still, moving, walking slow (<100 steps per minute) and fast (≥100 steps per minute (31)), running, cycling, stair climbing, and other physical activity (27,28). Times spent in sitting and lying were merged into SED. Standing still, moving, and walking slow were merged into LPA. Rest of the physical activity types were merged into MVPA. Diary-based information was used to determine sleep time as a period of time between going to and out of the bed. All 24-h movement behavior components were averaged across all valid days.

### Assessment of cardiometabolic biomarkers before and after retirement

A 10-h fasting blood samples were drawn by venipuncture in the morning shortly after the participants arrived to the clinical examination site. All blood samples were aliquoted at the same day and then stored at −80°C. All the analyses were performed at the laboratory of Turku University Hospital, Finland. Plasma cholesterol (total, LDL-cholesterol, HDL-cholesterol) and triglycerides were determined by enzymatic colorimetric tests (Cobas 8000 c702; Roche Diagnostics). Total/HDL-cholesterol ratio was calculated. C-reactive protein (CRP) was determined by nephelometric method (BN ProSpex/Atellica Neph, Siemens Healthineers). Fasting insulin was measured by electrochemiluminescence immunoassay method/Sandwich principle (Cobas 8000 e801; Roche Diagnostics), and fasting glucose was determined by enzymatic reference method with hexokinase. Fasting insulin could not be determined for 1 individual, leaving 211 individuals to the sample for fasting insulin.

### Assessment of preretirement characteristics

Sex, date of birth, and preretirement occupational title were obtained from the Keva Public Sector Pensions register. Participants were divided into two occupational status groups according to the occupational titles of the last known occupation preceding retirement by using the International Standard Classification of Occupations (ISCO) (32): manual workers (e.g., cleaners, maintenance workers to ISCO classes 5–9) and nonmanual workers (e.g., teachers, physicians, registered nurses, technicians to ISCO classes 1–4).

Other health-related characteristics were obtained from the questionnaire preceding the transition to retirement: smoking (no/yes), self-reported doctor-diagnosed chronic diseases (angina pectoris, myocardial infarction, cerebrovascular disease, claudication, osteoarthritis, osteoporosis, sciatica, fibromyalgia, rheumatoid arthritis, and diabetes) (no/yes, one or more), and mobility limitations as difficulties in walking 2.0 km (no/yes) (33,34).

### Statistical analyses

Descriptive information on participant characteristics is presented using means and SD for continuous variables and frequencies and percentages for categorical variables.

The 24-h movement behavior data were treated as compositional data, normalized to 24 h·d^−1^. The statistical analyses were conducted using R software (version 4.3.1; R Foundation for Statistical Computing, Vienna, Austria). An isometric logratio (ilr) transformation was used to map the compositional data into real-valued coordinates, which reduces the dimensionality of the data and allows standard statistical methods to be used (14). We used *pivot coordinates*, the specific type of *ilr* coordinates, that are a set of *ilrs* where the first coordinate enables one part of the composition (for instance, sleep) to be considered relative to the remaining parts of the composition (that is, SED, LPA and MVPA). We calculated four sets of pivot coordinates, that is, *ilrs*, and the change in *ilrs* by subtracting *ilrs* after retirement from *ilrs* before retirement to enable change in each behavior to be considered relative to the changes in the remaining behaviors.

We examined associations between change in each 24-h movement behavior in relation to the remaining behaviors and changes in cardiometabolic biomarkers during the transition to retirement using robust compositional regression models that are robust against the biased influence of outlying observations on the model fit (35,36). Cardiometabolic biomarkers were used as the dependent variable, and changes in 24-h movement behaviors (expressed as *ilrs*) as the independent variables. Covariates included 24-h movement behavior composition (expressed as *ilrs*) and cardiometabolic biomarkers before retirement, as well as age, sex, and occupational status. The model was repeated for each set of pivot coordinates. The associations were presented as beta coefficients and their 95% confidence intervals (CI). The beta coefficients indicate the change in dependent variable (e.g., mmol·L^−1^) for each 1-unit *ilr* increase, thus pointing out to presence of association, but effect sizes cannot be drawn directly from the beta coefficients.

To aid interpretation of the findings and to calculate effect sizes, the effect of observed reallocations between 24-h movement behaviors on cardiometabolic biomarkers was illustrated using the compositional isotemporal substitution model (11,37). First, systematic reallocations between movement behaviors were calculated based on the mean composition before retirement (8.3 h sleep, 9.7 h SED, 4.7 h LPA and 78 min MVPA). The size of the reallocations was chosen based on the observed actual range of change in MVPA, between −60 and 60 min. The 60-min size of reallocations was used also for reallocations between sleep, SED, and LPA to aid comparison of effect sizes between one-to-one reallocations between behaviors (e.g., to compare effect sizes between reallocating time from SED to MVPA vs SED to LPA). These reallocated compositions were then transformed to *ilrs* using the method explained previously. Second, the regression-based coefficients were applied on the calculated change in *ilrs* to predict changes in cardiometabolic biomarkers corresponding to changes in composition of 24-h movement behaviors during transition from work to retirement. Following the procedure applied in previous studies (37,38), the observed change in cardiometabolic biomarkers associated with no change in 24-h movement behavior composition was subtracted from the predicted changes in cardiometabolic biomarkers. We did this to isolate the effects of one-to-one reallocations between 24-h movement behaviors only (37,38). The results are shown as estimated changes in cardiometabolic biomarkers and their 95% CI. When the CI did not cover 0, the change was considered as significant. Example R code used for robust regression models and compositional isotemporal substitution analysis is provided in Supplemental Digital Content 2, http://links.lww.com/MSS/C987.

Finally, as a sensitivity analysis, we examined the possible seasonal effects on the associations between changes in movement behaviors and changes in cardiometabolic biomarkers. This was done by additionally adjusting for season (winter/spring/summer/autumn) at the preretirement measurement and follow-up time in days in the linear regression models. We also conducted a sensitivity analysis by excluding participants whose medication potentially affecting biomarker values changed during the follow-up. These were diabetes medication (ATC code A10), cholesterol medication (C10), and medications possibly affecting CRP values, that is, sex hormones (G03), corticosteroids (H02), antibacterials (J01), immunosuppressants (L04), anti-inflammatory and antirheumatic products (M01), and analgesics (N02) (change in diabetes medication: *n* = 5, 2.4%; change in cholesterol medication: *n* = 18, 8.5%; change in medication possibly affecting CRP values: *n* = 48, 23%). Moreover, given that associations between changes in 24-h movement behaviors and cardiometabolic biomarkers may differ depending on whether sleep is increased/decreased from insufficient or sufficient level (6–8), we conducted a sensitivity analysis by excluding those reporting more than 9 h of sleep per night before retirement (*n* = 24, 11%) from the robust regression models.

## RESULTS

Characteristics of the study population are presented in Table 1. The study population consisted mainly of women (82%) and nonmanual workers (70%). Before retirement, participants spent, on average, 8.3 h sleeping, 9.7 h sedentary, 4.7 h in LPA, and 78 min in MVPA per day.

**TABLE 1:** Characteristics of the study population (*n* = 212) before and after retirement.

	Before Retirement	After Retirement
Characteristics		
Age, mean (SD), yr	63.5 (1.1)	64.6 (1.2)
Women, *n* (%)	174 (82)	
Occupational group, *n* (%)		
Manual	64 (30)	
Nonmanual	148 (70)	
Current smoking, *n* (%)	9 (5)	8 (4)
Chronic diseases, *n* (%)	133 (67)	140 (69)
Mobility limitation, *n* (%)	17 (8)	17 (8)
Anthropometrics		
Body mass index, mean (SD), kg·m^−2^	26.3 (4.8)	26.2 (4.8)
Waist circumference, mean (SD), cm	91.5 (13.0)	90.6 (13.3)
Cardiometabolic biomarkers		
LDL-cholesterol, mean (SD), mmol·L^−1^	3.38 (0.88)	3.52 (1.04)
HDL-cholesterol, mean (SD), mmol·L^−1^	1.76 (0.50)	1.73 (0.47)
Total/HDL-cholesterol ratio, mean (SD)	3.47 (1.00)	3.53 (1.06)
Triglycerides, mean (SD), mmol·L^−1^	1.26 (0.54)	1.31 (0.53)
CRP, mean (SD), mg·L^−1^	1.50 (1.94)	1.67 (2.41)
Fasting insulin, mean (SD), mU·L^−1^	9.43 (8.10)	9.08 (7.36)
Fasting glucose, mean (SD), mmol·L^−1^	5.57 (0.91)	5.48 (1.30)
Accelerometer measurements		
No. valid measurement days (range)	4.6 (3–10)	4.6 (3–7)
No. daily log-determined nights (range)	3.1 (1–8)	3.2 (2–6)
Wear time during waking hours, h (IQR)	15.5 (15.0–16.1)	15.2 (14.6–15.7)
Compositional mean of sleep, SED, LPA, and MVPA, min	496, 583, 283, 78	520, 573, 271, 75

BMI, body mass index; IQR, interquartile range.

### Lipids

**FIGURE 1:**
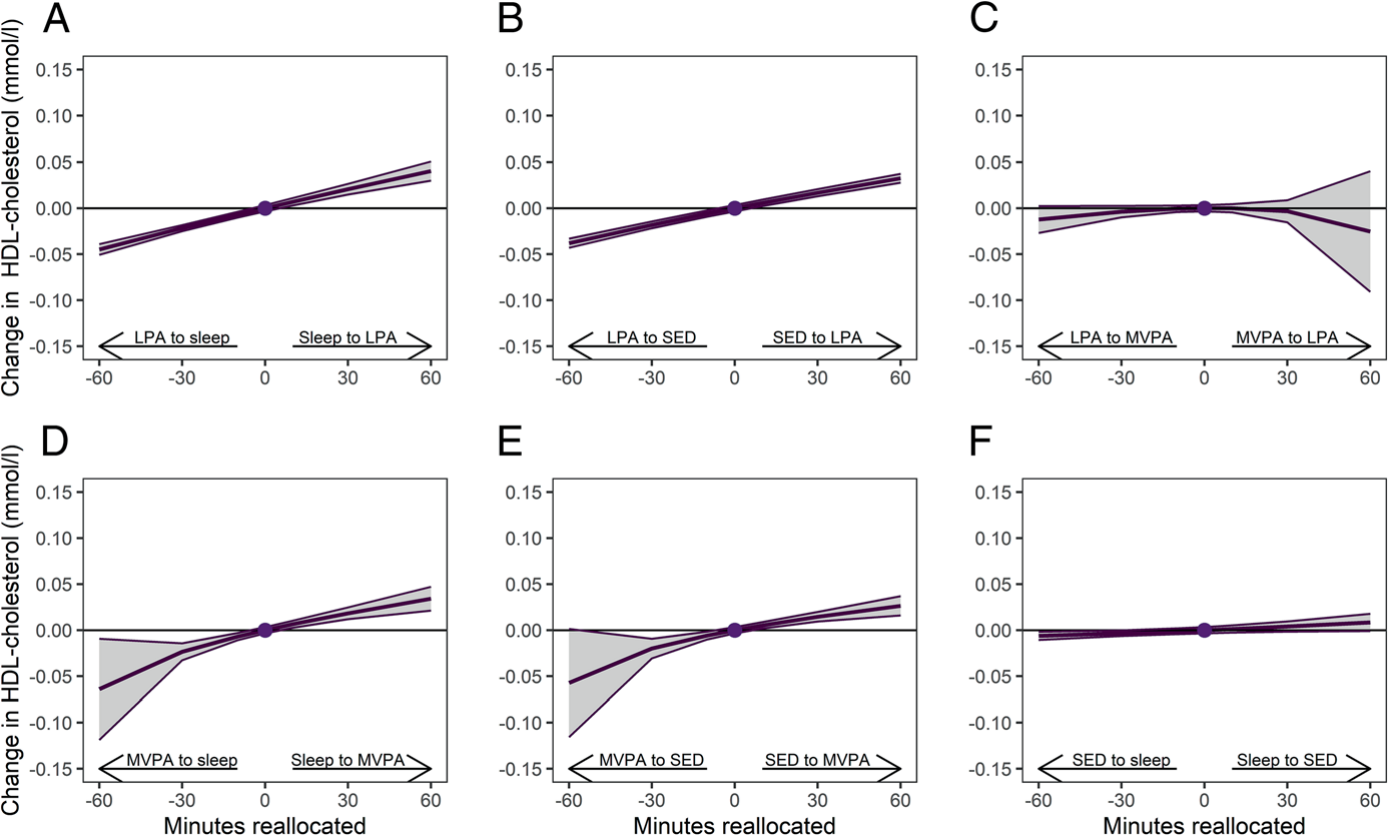
One-to-one reallocations between 24-h movement behaviors and changes in HDL-cholesterol over 1 yr from work to retirement. The dot at 0 indicates the mean preretirement composition of 8.3 h sleep, 9.7 h SED, 4.7 h LPA, and 78 min MVPA and HDL-cholesterol of 1.76 mmol·L^−1^.

**FIGURE 2:**
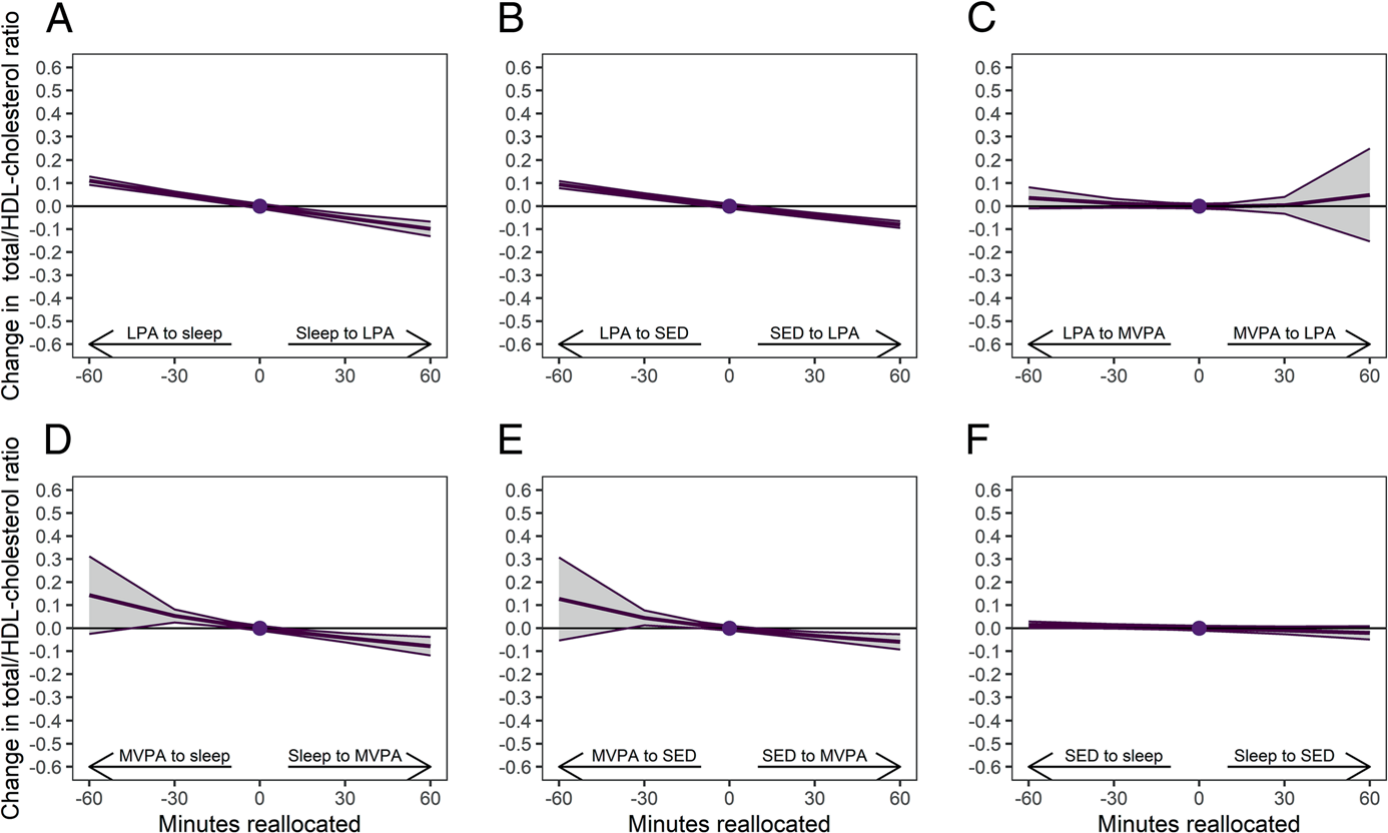
One-to-one reallocations between 24-h movement behaviors and changes in total/HDL-cholesterol ratio over 1 yr from work to retirement. The dot at 0 indicates the mean preretirement composition of 8.3 h sleep, 9.7 h SED, 4.7 h LPA, and 78 min MVPA and total/HDL cholesterol ratio of 3.47.

Examination of changes in each 24-h movement behavior in relation to the remaining behaviors indicated that increasing LPA in relation to the remaining behaviors was associated with an increase in HDL-cholesterol and decrease in total/HDL-cholesterol ratio and LDL-cholesterol (Supplemental Table 1, Supplemental Digital Content 1, Associations between changes in 24-h movement behaviors and changes in cardiometabolic biomarkers, http://links.lww.com/MSS/C986). For instance, reallocation of 30 min from sleep to LPA was associated with an increased HDL-cholesterol by 0.02 mmol·L^−1^ (95% CI, 0.01–0.03 mmol·L^−1^) (Fig. 1a), decreased total/HDL-cholesterol ratio by 0.05 (95% CI, −0.07 to −0.03) (Fig. 2a), and decreased LDL-cholesterol by 0.06 mmol·L^−1^ (95% CI, −0.07 to −0.04 mmol·L^−1^) (Supplemental Fig. 2a, Supplemental Digital Content 1, One-to-one reallocations between 24-h movement behaviors and changes in LDL-cholesterol over 1 yr from work to retirement, http://links.lww.com/MSS/C986). Reallocation of time between LPA and SED followed similar patterns as seen between LPA and sleep, but the effects were slightly smaller (Figs. 1b, 2b; Supplemental Figure 2b, Supplemental Digital Content 1, http://links.lww.com/MSS/C986). Increasing MVPA was also associated with an increase in HDL-cholesterol and decrease in total/HDL-cholesterol ratio, if it replaced sleep or SED (Figs. 1d, e; 2d, e). The effects of reallocation of 30 min from sleep to MVPA were relatively similar when compared with reallocation of 30 min from sleep to LPA.

**FIGURE 3:**
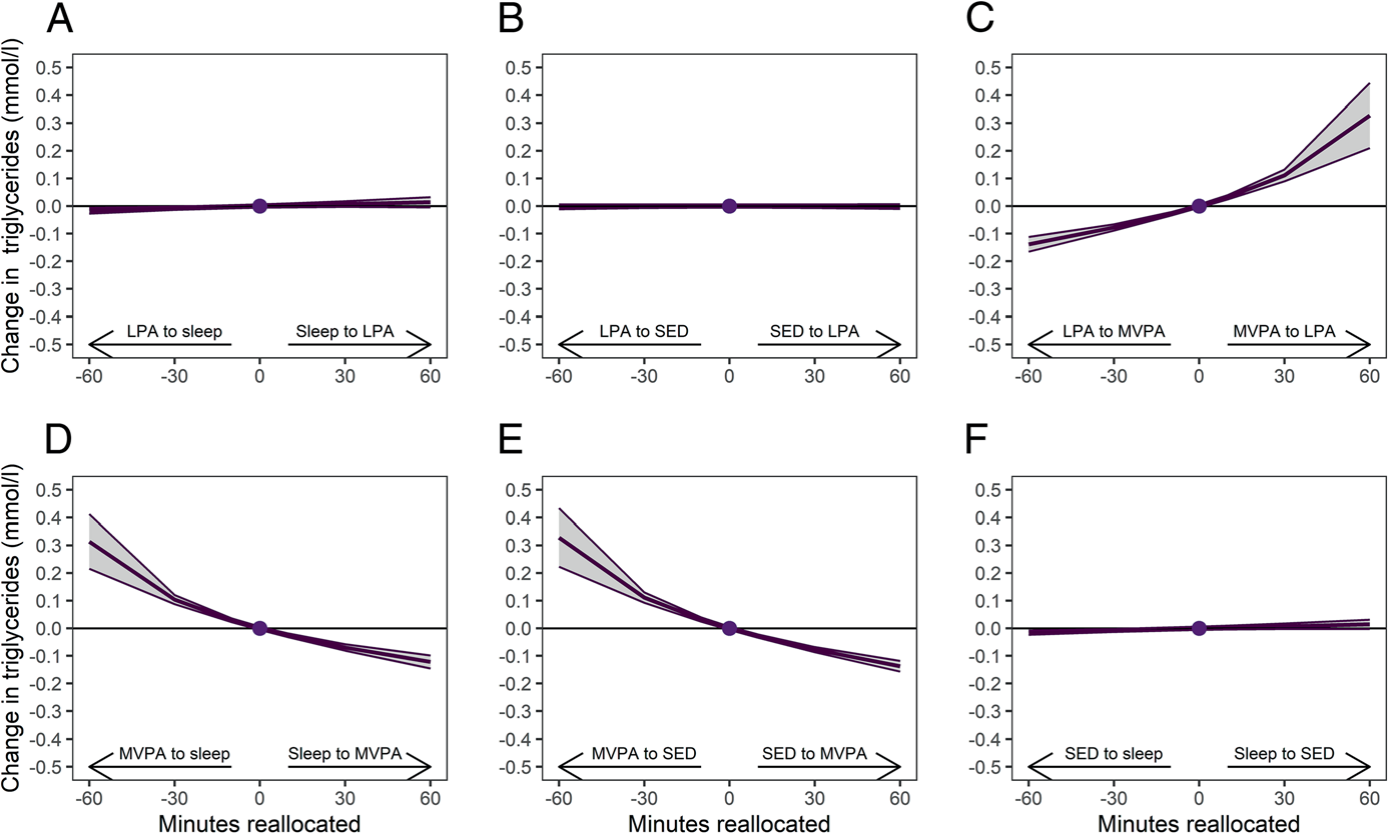
One-to-one reallocations between 24-h movement behaviors and changes in triglycerides over 1 yr from work to retirement. The dot at 0 indicates the mean preretirement composition of 8.3 h sleep, 9.7 h SED, 4.7 h LPA, and 78 min MVPA and triglycerides of 1.26 mmol·L^−1^.

Increasing MVPA in relation to the remaining behaviors was associated with a decrease in triglycerides (Supplemental Table 1, Supplemental Digital Content 1, http://links.lww.com/MSS/C986). As shown in Figures 3c–e, the effect of increasing MVPA did not markedly differ depending on which behavior MVPA replaced or was replaced with. For instance, replacing 30 min of sleep, SED, or LPA with MVPA was associated with a decrease in triglycerides by 0.07–0.08 mmol·L^−1^. In contrast, replacing 30 min of MVPA with sleep, SED, or LPA was associated with an increase in triglycerides by 0.10–0.11 mmol·L^−1^.

### C-reactive protein

Increasing SED in relation to the remaining behaviors tended to associate with an increase in CRP (Supplemental Table 1, Supplemental Digital Content 1, http://links.lww.com/MSS/C986). However, one-to-one reallocations between SED and the remaining behaviors resulted only in small and mostly nonsignificant changes in CRP (Supplemental Figs. 3b, e, and f, Supplemental Digital Content 1, One-to-one reallocations between 24-h movement behaviors and changes in CRP over 1 yr from work to retirement, http://links.lww.com/MSS/C986).

### Fasting insulin and glucose

Examination of changes in each 24-h movement behavior in relation to the remaining behaviors did not indicate significant associations for fasting insulin or glucose (Supplemental Table 1, Supplemental Digital Content 1, http://links.lww.com/MSS/C986). However, examining the isolated effect of one-to-one reallocations between MVPA and the remaining behaviors yielded decreases in fasting insulin (Supplemental Fig. 4, Supplemental Digital Content 1, One-to-one reallocations between 24-h movement behaviors and changes in fasting insulin over 1 yr from work to retirement, http://links.lww.com/MSS/C986). For instance, reallocation of 30 min from sleep, SED, or LPA to MVPA was associated with, on average, 0.50 mU·L^−1^ decrease in fasting insulin (Supplemental Figs. 4c–e, Supplemental Digital Content 1, http://links.lww.com/MSS/C986). Reallocations were not associated with changes in fasting glucose (Supplemental Fig. 5, Supplemental Digital Content 1, One-to-one reallocations between 24-h movement behaviors and changes in fasting glucose over 1 yr from work to retirement, http://links.lww.com/MSS/C986).

### Sensitivity analyses

Additional adjustments for the season at preretirement measurement and follow-up time did not notably change the results (Supplemental Table 2, Supplemental Digital Content 1, Associations between changes in 24-h movement behaviors and changes in cardiometabolic biomarkers by taking into account measurement season and follow-up time, http://links.lww.com/MSS/C986). When excluding those participants whose medication (cholesterol/anti-inflammatory) changed during the follow-up, the associations between LPA and LDL-cholesterol and SED and CRP attenuated (Supplemental Table 3, Supplemental Digital Content 1, Associations between changes in 24-h movement behaviors and changes in cardiometabolic biomarkers by taking into account changes in medication, http://links.lww.com/MSS/C986). Moreover, after excluding long-sleepers (>9 h), findings regarding fasting glucose changed; increasing LPA tended to associate with an increase in fasting glucose, whereas the opposite was observed for increasing sleep (Supplemental Table 4, Supplemental Digital Content 1, Associations between changes in 24-h movement behaviors and changes in cardiometabolic biomarkers without long sleepers, http://links.lww.com/MSS/C986).

## DISCUSSION

In this study, we utilized a natural experiment setting and repeated measurements across the retirement transition as well as the CoDA methodology to examine longitudinal associations between changes in 24-h movement behaviors and concurrent changes in cardiometabolic biomarkers. Our findings indicated clear beneficial associations between higher LPA and MVPA in relation to sleep and SED and HDL-cholesterol, total/HDL-cholesterol ratio, and triglycerides. Findings related to LDL-cholesterol, CRP, fasting insulin, and glucose were less conclusive—mainly affected by preretirement sleep duration and/or concurrent changes in medication.

Our findings add to the previous experimental evidence highlighting the benefits of aerobic exercise for improving HDL-cholesterol (39,40) by showing that increasing physical activity at any intensity (LPA and MVPA) at the expense of more passive behaviors is associated with increased HDL-cholesterol and decreased total/HDL-cholesterol ratio. As little as 10-min increase in LPA (including standing still, moving, and slow walking) at the cost of passive behaviors yielded a significant increase in HDL-cholesterol (based on 95% CIs). Although not measured in the present study, the mechanisms underlying our observations are likely explained by physical activity–induced increased activity of lipoprotein lipase and other enzymes, which enhances conversion of the VLDL particles to HDL particles, increases ester transfer to HDL particles, and decreases transfer of HDL to other lipoproteins (39,41). Previous CoDA-based studies have been cross-sectional (9,16–18), which limits comparison between our results on within-individual changes and previous findings on between-individual differences. These studies have mainly reported associations between higher MVPA in relation to the remaining behaviors and higher HDL-cholesterol (9,16–18), whereas associations for LPA have been mostly nonsignificant (9,16,18). In addition to the differences in the study design, the methods estimating LPA vary across studies (threshold-based method capturing basically light nonstationary behaviors [42,43] vs posture-based method capturing standing and light upright activities as LPA), which further limits comparisons between findings.

Our results corroborate with previous literature showing that physical activity of higher intensities is needed to reduce triglycerides (39). We found a clear association between increasing MVPA (including fast walking, stair walking, cycling, or running) in relation to the remaining behaviors and decrease in triglycerides. These findings are consistent with previous cross-sectional CoDA-based studies (9,15–17). We observed that a decrease in MVPA was associated with a more substantial increase in triglyceride levels compared with the decrease in triglyceride levels observed when MVPA increased. This asymmetric association is a common finding in the literature (9,17,44) and consistent with exercise physiology principles indicating that dropping physical activity levels leads to deconditioning rapidly, whereas improving health/fitness by exercise overload takes much more time (9,45,46). Our findings also suggest that the effect of changing MVPA does not depend on which activity MVPA replaces or is replaced with. The mechanism underlying these observations is likely explained by increased muscle activity that increases enzymatic activity, leading to increased uptake and use of circulating triglycerides in muscle tissues (41).

We found an indication of an association between increasing LPA, but not MVPA, in relation to the remaining behaviors and a decrease in LDL-cholesterol. However, this association attenuated after taking into account concurrent changes in medication and preretirement sleep duration. Our findings are consistent with experimental evidence that has demonstrated how physical activity primarily improves HDL-cholesterol and triglyceride levels, whereas improvements in LDL-cholesterol have been mostly nonsignificant (47), at least without dietary modifications (48).

The observed effect sizes for lipids can be compared with results obtained from exercise intervention studies. Based on a recent meta-analysis (49), adding 2–3 h of supervised running exercise (MVPA) per week, equivalent to ≈17–25 min running per day, increased HDL-cholesterol by 0.09 mmol·L^−1^ and decreased triglycerides by 0.19 mmol·L^−1^ over 1 yr. In our study, the effects of replacing SED or sleep with LPA or MVPA were, on average, 20% to 40% of the effect of running interventions. These markedly smaller effect sizes are understandable because there is considerably more heterogeneity in changes in physical activity during life transitions (e.g., some increase, others decrease their physical activity) compared with experimental studies that aim to systematically increase physical activity over a short intervention period (usually some weeks) among study participants. It should also be noted that the co-dependency between 24-h movement behaviors was not taken into account in the studies included in the meta-analysis.

We found an association between increasing SED in relation to the remaining behaviors and increasing CRP levels, but effect sizes were small and affected by concurrent changes in anti-inflammatory medication. The proportion of those whose medication affecting inflammatory markers changed during the follow-up was relatively high (23%); thus, excluding these participants in a sensitivity analysis also reduced statistical power significantly. We did not observe notable reductions in CRP levels when physical activity replaced SED, which is conflicting with findings from cross-sectional CoDA-based studies (9,16,50), or previously observed detrimental associations for sleep (9,16). Future studies with larger study samples are needed to elaborate the relationships between changes in 24-h movement behaviors and inflammatory markers.

Our findings suggest that increasing MVPA at the cost of sleep, SED, or LPA may reduce fasting insulin levels during 1 yr across the retirement transition. The associations were apparent only when examining the isolated effect of one-to-one reallocations (37,38) between MVPA and sleep, SED, and LPA. These findings are in line with the previous cross-sectional CoDA-based studies (9,16,17,19) and experimental evidence showing beneficial effects of aerobic exercise on fasting insulin levels (51). We did not observe associations for fasting glucose, which is conflicting with previous cross-sectional CoDA-bases studies (9,16,17,19), but in line with a previous meta-analysis among general population (51). Effects of physical activity on fasting glucose have mostly been reported among subclinical and clinical study populations (52,53). Moreover, previous experimental evidence has mainly demonstrated acute beneficial effects of aerobic exercise (52,54), high-intensity interval training (51), resistance training (53), and breaking up prolonged SED with physical activity (55–60) on postprandial glucose, which may be more prone to exercise-induced changes compared with fasting glucose (61).

Based on our findings, sleep seemed to be rather neutral or even negative behavior with respect to changes in cardiometabolic biomarkers. The effects of increasing sleep versus SED at the expense of physical activity were relatively similar, and no improvements in cardiometabolic biomarkers were observed when sleep replaced SED. Increasing sleep from insufficient level generally improves cardiometabolic health (62), but this may not be the case when sleep is already at an adequate level. Increasing sleep from a sufficient level takes time away from behaviors with higher energy expenditure and reduces the use of glucose and lipids as an energy source, which may lead to negative changes in cardiometabolic biomarkers. However, we observed that after excluding long sleepers, associations changed so that increasing LPA seemed to be more negative and increasing sleep more positive for fasting glucose, suggesting that associations may depend on baseline sleep duration. Given that sleep duration seemed to be at a sufficient level among most of the retirees at baseline (7–9 h per night), and there were very few short (14%) or long sleepers (11%), the role of baseline sleep duration should be elaborated more in larger study samples with higher proportion of short and long sleepers.

Our study has several clinical implications. Based on our previous studies, after exiting working life, most retirees increase sleep duration, which is reflected mainly as decreased physical activity levels, whereas sedentary time remains at high level (21). Given that increasing sleep at the cost of physical activity seems to be associated with negative changes lipid profile, yet increased sleep may be beneficial for some retirees (e.g., for those with insufficient sleep) (62), it is important to ensure that longer sleep does not decrease physical activity levels. Simply moving more and sitting less throughout the day could improve cardiometabolic health by improving cholesterol levels, additional higher-intensity physical activity may be needed to elicit improvements in other lipids, mostly triglycerides. Therefore, it seems essential to encourage retirees to maintain or increase physical activity, and emphasize the importance of a balanced daily routine after retirement.

Given that the negative effects of reducing MVPA were stronger when compared with positive effects of increasing MVPA, maintaining MVPA levels after retirement could be a reasonable intervention goal among recent retirees. However, it should be acknowledged that, especially for those individuals whose preretirement activity primarily stems from physically active work and active commuting, special efforts may be required to maintain their activity levels after retirement. This can be achieved through activities such as household chores, taking active breaks to break up prolonged sitting, choosing active modes of transportation, establishing new physical activity routines, and participating in sports. Whenever possible, adding targeted or structured exercise programs to ensure adequate intensity should also be recommended.

Strengths of the current study include a longitudinal study design and repeated accelerometer-based measurements, which are not subject to recall and information bias such as commonly used self-reports (63). Moreover, we used posture-based identification of SED, LPA, and MVPA with ActiPASS (Acti4), which has shown to identify postures and physical activities with high sensitivity (80%) and specificity (>90%) during semistandardized and free-living conditions (28,29). In addition, we used state-of-the-art statistical methods to examine changes and reallocations between codependent components of a 24-h day (11).

The main limitation is the fact that we examined concurrent short-term changes in 24-h movement behaviors and cardiometabolic biomarkers; thus, we were unable to show the direction of causation. The relatively small study sample increases the risk of type II errors (failure to identify significant effect that actually exists) (64). In small sample sizes, the potential influence of outlying observations is also greater when compared with larger study samples. However, we used robust regression models to minimize influence of outlying observations (35,36). We did not have information of participant’s diet, which may have an effect on cardiometabolic biomarkers and which may also be subject to changes when transitioning from work to retirement (65). Moreover, we were unable to separate strength training, which also improves lipid profile (39), from the physical activity estimates. In our study, some strength training activities done sitting may have been misclassified as SED. Sleep time was based on self-reported measures that reflect time in bed rather than actual sleep time. Moreover, because we did not have information on sleep efficiency, it remains unclear how much of the increased sleep time represents actual sleep versus how much of it consists of lying in bed awake, that is, sedentary time. Thus, sleep efficiency and/or other sleep quality measures should be taken into account in future studies. Finally, given that the current study population was generally leaner, healthier, and more active compared with the survey-only study participants (22), the generalizability of the findings may be limited.

## CONCLUSIONS

Increasing LPA and MVPA at the cost of sleep and SED was associated with some improvements in especially blood lipid profile during the transition from work to retirement. Thus, promoting physical activity at the cost of more passive behaviors may improve cardiometabolic health during the transition to retirement. However, future studies with longer follow-up are needed to elaborate long-term health effects of changes in 24-h movement behaviors.
